# Supplementary material for: Appraising the Welfare of Thoroughbred Racehorses in Training in Queensland, Australia: The Incidence, Risk Factors and Outcomes for Horses after Retirement from Racing
Source: Animals (Basel). 2021 Jan 11;11(1):142. doi: 10.3390/ani11010142 (PMC7827103; doi:10.3390/ani11010142)
Supplement: Supplementary file 1 [file animals-11-00142-s001.pdf]

animals

Appraising the Welfare of Thoroughbred Racehorses in Training in Queensland, Australia: The Incidence, Risk Factors and Outcomes for Horses after Retirement from Racing

Kylie L. Crawford, Anna Finnane, Ristan M. Greer, Clive J. C. Phillips, Solomon M. Woldeyohannes, Nigel R. Perkins and Benjamin J. Ahern

Supplementary Materials:

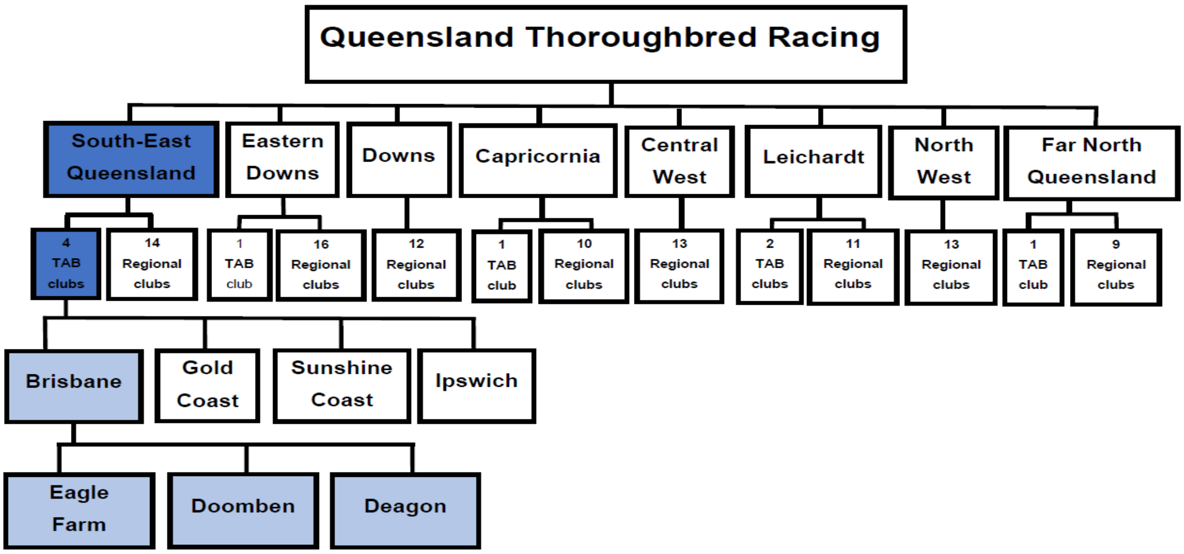

Figure S1. The structure of the Queensland Thoroughbred Racing Industry and the TAB (Totalisator Agency Board) and regional clubs for each region. The target population for our study is the TAB clubs of South East Queensland (dark blue), and our sampling frame is Brisbane Racing Club (light blue).

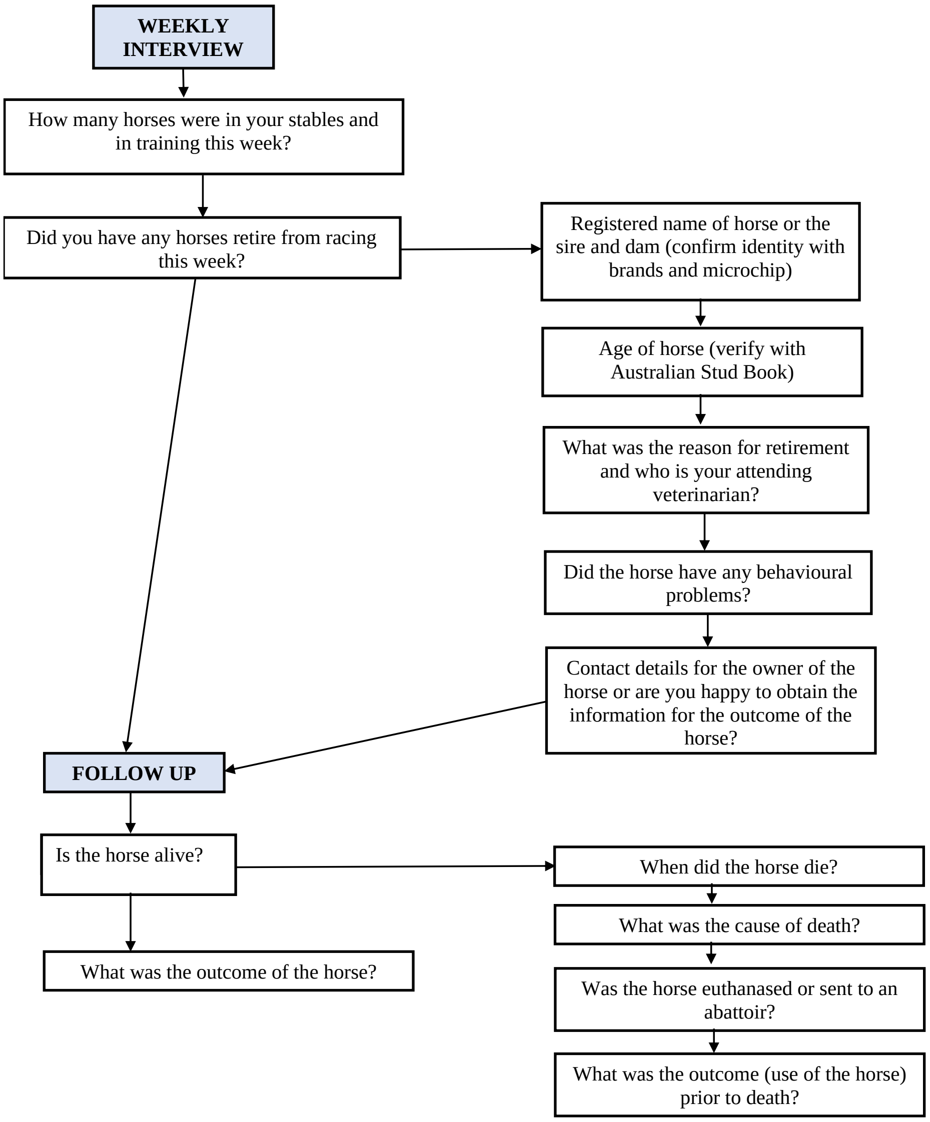

Figure S2. Structured weekly interview with interview with trainers or foreperson-Prospective survey of retired racehorse
